# Gene profiling and serotyping of multidrug-resistant *Listeria monocytogenes* isolated from humans, animals, and dairy products

**DOI:** 10.1186/s12917-025-05138-4

**Published:** 2025-11-22

**Authors:** Sabah Ibrahim Shaaban, Noha Awwad, Mohamed Nossair, Mousa Ayoub, Abd El-Majeed Eid

**Affiliations:** 1 Department of Zoonoses, Faculty of Veterinary Medicine, Damanhour University, Damanhour, Egypt; 2 Department of Bacteriology, Immunology and Mycology, Faculty of Veterinary Medicine, Damanhour University, Damanhour, Egypt; 3 Department of Animal Hygiene and Zoonoses, Faculty of Veterinary Medicine, Alexandria University, Alexandria, Egypt; 4 Department of Animal Hygiene, Faculty of Veterinary Medicine, Damanhour University, Damanhour, Egypt; 5 Matrouh Veterinary Directorate, Damanhour University, Damanhour, Egypt

**Keywords:** Listeria monocytogenes, Antimicrobial resistance, Serotyping, Molecular characterization, Dairy products, Farm animals, Zoonoses

## Abstract

**Background:**

*Listeria monocytogenes* is a foodborne pathogen of a major public health concern due to its ability to cause severe illness and its increasing antimicrobial resistance. This study aimed to determine the prevalence, antimicrobial susceptibility, serotype distribution, and virulence genes profiles of *L. monocytogenes* isolated from food products, farm animals, and humans in Egypt.

**Methodology:**

A total of 750 samples were collected, including dairy products, raw meat, animal fecal samples, aborted fetuses, and human stool samples. Isolation and identification were performed according to ISO, (2022) followed by serotyping and molecular characterization of virulence genes (*iap*, *hlyA*, and *actA*). Antimicrobial susceptibility testing was conducted using the disk diffusion method according to CLSI guidelines.

**Results:**

*Listeria spp.* were detected in 18.5% of food products, 4.75% of animal fecal samples, 18.0% of aborted fetuses, and 17.0% of human samples. The highest contamination rates were observed in Kareish cheese (28.0%) and raw milk (22.0%). Serotype 4b was the most prevalent among isolates. All isolates harbored the *iap* gene, while the *hlyA* and *actA* genes were detected in 80% and 67% of isolates, respectively. Alarmingly, all isolates were resistant to clindamycin and nalidixic acid, and high resistance rates were observed against penicillin (93.3%) and ampicillin (86.7%), although imipenem and amikacin remained effective.

**Conclusion:**

These findings highlight the circulation of multidrug-resistant and virulent *L. monocytogenes* strains in Egypt’s food chain and among occupational groups. Enhanced hygiene measures, continuous surveillance, and prudent antibiotic use are urgently needed to mitigate the associated public health risks.

**Supplementary Information:**

The online version contains supplementary material available at 10.1186/s12917-025-05138-4.

## Introduction

*Listeria monocytogenes* is a foodborne pathogen that causes severe illness and poses a significant risk to food safety. It is responsible for listeriosis, a foodborne illness with up to a 30% death rate, which positions it as one of the deadliest foodborne pathogens [1]. Its ability to survive and multiply in cold, high-salt, and acidic environments presents a major challenge to the food processing and storage industries [2]. The detection of *Listeria* in food, livestock, and human samples points to the intricate and widespread transmission occurring within the food production system [3]. *Listeria* can mainly be found in dairy products and raw meat. Increased concern is directed toward ready-to-eat meals because they do not undergo further heating during consumption [4]. Soft cheeses have been associated with numerous multinational listeriosis outbreaks, contributing to 15% of the outbreaks monitored in the dairy sector from 2020 to 2022 [5]. Recently, the rising antimicrobial resistance in *L. monocytogenes* has become a heightened concern in the public health field. Until recently, *L. monocytogenes* was believed to be one of the “safer” pathogens, as it was susceptible to most antibiotics. However, mounting evidence suggests that resistance in *L. monocytogenes*, especially to bacterial xenobiotics, has been on the rise. This has been documented in food sources [6]. There are increasing reports of resistance to β-lactam antibiotics like penicillin and ampicillin, which were once used as first-line therapy medications [7]. This alarming trend has been caused by a horizontal gene transfer and the use of antibiotics in food production [8]. It is crucial to determine the serotype distribution in foods, as this allows for risk assessment and investigation of foodborne outbreaks [9]. Serotyping remains a vital epidemiological tool for *L. monocytogenes*, with serotypes 1/2a, 1/2b, 1/2c, and 4b representing over 95% of the worldwide human cases of listeriosis [10]. These serotypes are not only critical in epidemiological tracing but also correlate with virulence potential [7]. Evidence of genetic diversity is provided by molecular characterization of the strains obtained from food, animal, and human samples, which are noted to have varied and sometimes distinct genetic differences, suggesting regional differences in strain circulation and selection [11]. The basic principles of food microbiology are also a critical foundation for understanding these contamination risks [12]. So, this study aimed to determine the prevalence, antimicrobial susceptibility, serotype distribution, and virulence genes profiles of *L. monocytogenes* isolated from food products, farm animals, and humans in Egypt.

## Materials and methods

### Study area and design

 The present study was conducted from January 2023 to June 2024 at El-Beheira Governorate, Egypt, to determine the prevalence, antimicrobial susceptibility, serotype distribution, and virulence gene carriage of *Listeria monocytogenes* from food products, farm animals, and humans in the study area. The target sample size was based on a prevalence-estimation formula for proportions: Z^2^ P (1 − P)/d^2^. Assuming 5% expected prevalence, 3% precision, and 95% confidence, the minimum required sample size was 203 per stratum. To increase precision, a total of 750 samples were collected, following approaches applied in similar studies [13, 14].

### Sampling

 A total of 750 samples were collected from different sources in El-Beheira governorate, Egypt. The samples included, Food products (*n* = 200): raw milk (*n* = 50), yogurt (*n* = 50), Kareish cheese (*n* = 50), and raw meat (*n* = 50), randomly purchased from supermarkets. Animal samples (*n* = 400): fecal samples from cattle (*n* = 100), buffaloes (*n* = 100), sheep (*n* = 100), and goats (*n* = 100) were collected directly from the rectum using sterile swabs (100 per species). Aborted fetal tissues (*n* = 50): obtained from private farms with owners’ permission (25 bovine, 25 ovine), and Human samples (*n* = 100): stool samples collected from farm workers (*n* = 50) and food handlers (*n* = 50).

Food and aborted fetal samples were separately placed in sterile ice-packed containers and transported immediately to the laboratory for processing within 6 h of collection. Swabs and stool samples were placed in sterile transport medium and processed on the same day [15].

### Isolation of *Listeria* spp

 The isolation of *Listeria monocytogenes* and *Listeria* spp. was carried out according to the ISO, (2022) horizontal method for detection [16], with minor modifications adapted to the sample type. This method is considered a gold standard and has been validated in other studies for various food matrices [17]. Food samples (25 g or mL; milk, yogurt, cheese, meat): Each sample was aseptically transferred into 225 mL half-Fraser broth for primary enrichment (30 °C, 24 ± 2 h), followed by secondary enrichment in Fraser broth (37 °C, 48 ± 3 h). Cultures were streaked onto PALCAM and Oxford agars for colony isolation. Fecal and swab samples (1–2 g or one swab): Inoculated into 10 mL half-Fraser broth and incubated under the same conditions. Secondary enrichment and selective plating were identical to food samples. Tissue samples (10 g; aborted fetuses and organs): Homogenized in 90 mL half-Fraser broth then processed as for food samples. Using 10 g instead of 25 g was chosen to accommodate the limited size of tissue samples and to optimize recovery from organ matrices.

Typical colonies from selective agars were picked up for confirmation. All media (half-Fraser/Fraser broths, PALCAM and Oxford agars, and TSA/TSB) and selective supplements were obtained from Oxoid (Basingstoke, UK).

### Identification of *Listeria* spp

 After incubation, colonies with typical morphology (e.g., gray-green colonies with black halos on Oxford; characteristic colonies on PALCAM) were picked. To ensure a representative sample and address potential variations in morphology, 2–3 colonies were chosen when morphotypes varied, while in most positive samples one representative colony was carried forward to avoid over-representation. If clearly distinct morphotypes were present, each was processed separately to detect mixed infections. Preliminary identification included Gram staining of pure cultures [18] and standard biochemical characterization: catalase test, motility at 25 °C, Esculin hydrolysis, β-hemolysis on blood agar, and carbohydrate fermentation (rhamnose, xylose, mannitol) [19, 20]. Biochemically identified isolates were sub cultured on tryptone soya yeast extract agar, and colonies were stored in glycerol at − 20 °C for future analysis. For further confirmation, a *Listeria* latex agglutination test kit (Oxoid, Basingstoke, UK) was applied in conjunction with the biochemical results [21].

### Antimicrobial susceptibility testing (AST)

 Susceptibility testing was performed by disk diffusion on Mueller-Hinton agar (Oxoid, UK), supplemented with 5% sheep blood when required, according to CLSI standards (M100, 32nd edition, 2022) [22]. Sixteen antibiotics representing major drug classes were tested, selected for clinical relevance and comparability with regional surveillance. Zone diameters were measured and interpreted using CLSI breakpoints. For agents without *Listeria*-specific criteria, results were reported cautiously with reference to related organisms or EUCAST. Quality control strains (*Escherichia coli* ATCC 25922, *Staphylococcus aureus* ATCC 25923, *Listeria monocytogenes* ATCC 19115) were used, and a multiple antibiotic resistance (MAR) index was calculated for each isolate.

### Serological typing

 Serological typing of *Listeria monocytogenes* isolates was performed using a commercial serotyping kit (Denka Seiken Co., Tokyo, Japan; antisera also available from Difco, Becton Dickinson, USA) according to the manufacturer’s instructions. The method is based on antibodies that specifically react with somatic (O) and flagellar (H) antigens [23].

### Molecular characterization DNA Extraction and Amplification

 Genomic DNA was extracted from pure cultures of presumptive *Listeria* isolates using the GeneJET Genomic DNA Purification Kit (Fermentas, Lithuania) [24]. PCR assays targeted *Listeria* spp., *L. monocytogenes*, virulence genes (iap, hlyA, actA), and selected serotypes, using published primers (Table 1). Multiplex PCR for virulence genes was performed according to Rawool et al. [25]. Amplifications were conducted on a Master cycler (Eppendorf, Hamburg, Germany). Products were separated on 1.5% agarose gel in 1× TBE buffer, stained with ethidium bromide, and visualized under UV illumination. A 100 bp DNA ladder served as the molecular size marker [26].

**Table 1:** Primers used for detection of virulence genes (iap, hlyA, actA)

Target gene	Primer sequence (5′ → 3′)	Product size (bp)	Reference
*iap* (F)	**ACAAGCTGCACCTGTTGCAG**	**131**	[25]
*iap* (R)	**TGACAGCGTGTGTAGTAGCA**		
*hlyA* (F)	**GCAGTTGCAAGCGCTTGGAGTGAA**	**456**	[25]
*hlyA* (R)	**GCAACGTATCCTCCAGAGTGATCG**		
*actA* (F)	**CGCCGCGGAAATTAAAAAAAGA**	**839**	[25]
*actA* (R)	**ACGAAGGAACCGGGCTGCTAG**		

* *L. monocytogenes*, its virulence genes (as invasive associated protein (iap), haemolysin (hlyA) and actin polymerization protein (actA) genes)

#### Statistical analysis

Data were managed in Microsoft Excel and analyzed with SAS (SAS Institute Inc, Cary, NC, USA). Prevalence was expressed as proportions with 95% confidence intervals (CIs, Wilson method). Group differences were assessed using chi-square; *p value* < 0.05 was considered significant. The importance of *L. monocytogenes* occurrence among the examined samples was carried out according to SAS [27],.

## Results

### Overall prevalence of *Listeria* spp in different samples

Out of 750 samples examined, *Listeria spp*. were recovered from 82 (10.9%). Food products showed the highest isolation rate (18.5%, 37/200), followed by human samples (17.0%, 17/100), animal fecal samples (6.3%, 25/400), and aborted fetuses (8.0%, 4/50). The difference in prevalence across sources was statistically significant (χ² = 25.79, *p* < 0.001) **(**Table 2**)**.

**Table 2:** Prevalence of *Listeria spp.*In different sample sources (*n* = 750)

Source	Samples tested	Positive (*n*)	%	
Farm animals (feces)	400	19	4.75	
Aborted fetuses	50	9	18	
Food products	200	37	18.5	
Humans	100	17	17	
Total	750	82	10.9	
Chi² (p-value)				**χ² = 25.8 (p < 0.001)**

* Significant difference in prevalence across sources was found, with higher detection in food and human samples compared to animals.

### Isolation of *Listeria spp.* from food products

Among food samples, the overall prevalence in different food products (18.5%) **(**Table 2**)**, the highest prevalence was recorded in Kareish cheese (28.0%, 14/50), followed by raw milk (22.0%, 11/50), raw meat (16.0%, 8/50), and yogurt (8.0%, 4/50). Differences among food types were not statistically significant (*p* > 0.05) **(**Table 2**).**

### Isolation of *Listeria spp.* from animals and aborted fetuses

From 400 fecal samples, *Listeria* spp. were detected in 19 cases (4.75%), with cattle showing the highest rate (7.0%), followed by sheep (5.0%), buffaloes (4.0%), and goats (3.0%). No significant differences were observed among animal species (χ² = 1.93, *p* > 0.05). From aborted fetuses, 18.0% (9/50) were positive, with slightly higher detection in cattle (20.0%) compared to sheep (16.0%) **(**Table 2**)**.

### Isolation of *Listeria spp.* from humans

Among 100 stool samples collected from occupationally exposed groups, *Listeria* spp. were isolated from 17 (17.0%) **(**Table 2**)**, including 12 positive samples from farm workers (24.0%) and five from food handlers (10.0%). The distribution was not statistically different (*p* > 0.05). *L. monocytogenes* was the predominant species, recovered from six individuals (6.0%).

### Distribution of different *Listeria spp.* isolated from different sources

Across all sources, six *Listeria* species were identified: *L. monocytogenes*, *L. ivanovii*, *L. innocua*, *L. grayi*, *L. seeligeri*, and *L. welshimeri*. The predominant species were *L. innocua* (5.5%) and *L. ivanovii* (5.0%) in food, while *L. ivanovii* (1.5%) predominated in animals. Among human isolates, *L. monocytogenes* was most frequent (6.0%) (Table 3).

**Table 3:** Prevalence and species distribution of *Listeria* spp. in different sources (*n* = 750)

Species	Animals feces (*n* = 400)	Fetuses (*n* = 50)	Food (*n* = 200)	Humans (*n* = 100)	Total (*n* = 750)
*L. monocytogenes*	4 (1.0%)	5 (10.0%)	5 (2.5%)	6 (6.0%)	20 (2.7%)
*L. ivanovii*	6 (1.5%)	1 (2.0%)	10 (5.0%)	5 (5.0%)	22 (2.9%)
*L. innocua*	4 (1.0%)	2 (4.0%)	11 (5.5%)	3 (3.0%)	20 (2.7%)
*L. welshimeri*	1 (0.25%)	1 (2.0%)	5 (2.5%)	1 (1.0%)	8 (1.1%)
*L. grayi*	3 (0.75%)	0 (0.0%)	3 (1.5%)	2 (2.0%)	8 (1.1%)
*L. seeligeri*	1 (0.25%)	0 (0.0%)	3 (1.5%)	0 (0.0%)	4 (0.5%)
Total positive	19 (4.75%)	9 (18.0%)	37 (18.5%)	17 (17.0%)	82 (10.9%)
Chi² value					**p > 0.05**

* No significant association between species distribution and sample source, indicating random occurrence.

### Antimicrobial susceptibility 

The antimicrobial resistance profile of *L*. *monocytogenes* from animal sources showed alarming resistance rates (Table 4). All isolates presented 100% resistance to clindamycin and nalidixic acid, with high resistance to penicillin (92.9%) and ampicillin (85.7%). There are no statistically significant differences in antimicrobial resistance patterns between human isolates for most antibiotics, suggesting similar resistance mechanisms across sources. All isolates presented 100% resistance to clindamycin, nalidixic acid, penicillin and ampicillin, with high resistance to tetracycline (83.3%) and erythromycin, ciprofloxacin and linezolid (66.7%).

**Table 4:** Antimicrobial susceptibility of *L. monocytogenes*

Antimicrobial resistance	Isolates (*n*)	Resistant %
Clindamycin, Nalidixic acid	15	100
Penicillin	14	93.3
Ampicillin	13	86.7
Tetracycline	11	73.3
Erythromycin	10	66.7
Ciprofloxacin	6	40.0
Cefotaxime	5	33.3
Amikacin	2	13.3
Imipenem	1	6.7
^*^Chi² (AMR profiles)	χ² = 114.4	*p* < 0.001

* No significant differences between animal/food vs. human isolates (*p* > 0.05)

### Serotyping of *L. monocytogenes* isolates

Serotyping of *L. monocytogenes* revealed serotypes 1/2a, 1/2b, 1/2c, 3b, 3c, 4b, and 4 d, with 4b being the most prevalent across food, animal, and human samples (Table 5).

**Table 5:** Serotypes and antimicrobial resistance of *L. monocytogenes* isolates (*n* = 15).

Serotype distribution	Isolates No (%)
4b	8 (53.3)
1/2b	3 (20.0)
1/2a, 1/2c, 3b, 3c, 4d	4 (26.7)
Chi²	*p* > 0.05

*No significant difference was found, *p* > 0.05.

### Molecular characterization of different *listeria spp.* isolated from different sources

#### Characterization of Virulence genes of *listeria spp*

**Fig. 1:**
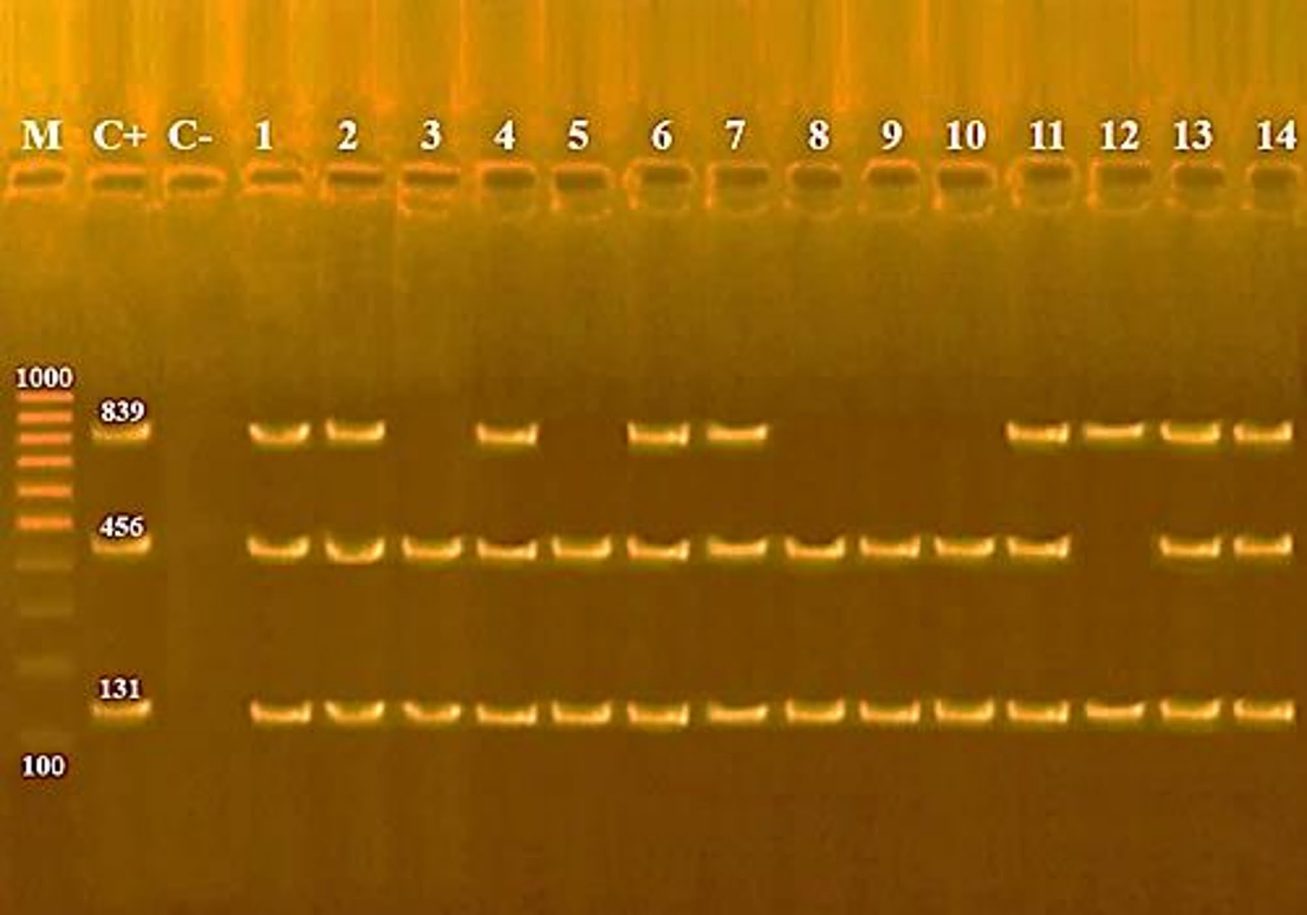
Agarose gel electrophoresis of multiplex PCR of *iap* (131 bp), *hylA* (456 bp) and *actA* (839 bp) genes for characterization of *Listeria monocytogenes* isolated from animal sources.(Lane M:100 bp ladder as molecular size DNA marker.Lane C+: Control positive *L.monocytogenes* for *iap*, *hylA* and *actA* genes. Lane C-: Control negative. Lanes 1, 2, 4, 6, 7, 11, 13 & 14: Positive strains for *iap*,* hylA* and *actA*genes. Lanes 3, 5, 8, 9 & 10: Positive *L. monocytogenes* strains for *iap *and *hylA* genes. Lane 12: Positive *L. monocytogenes* strain for *iap *and *actA *genes).

**Fig. 2:**
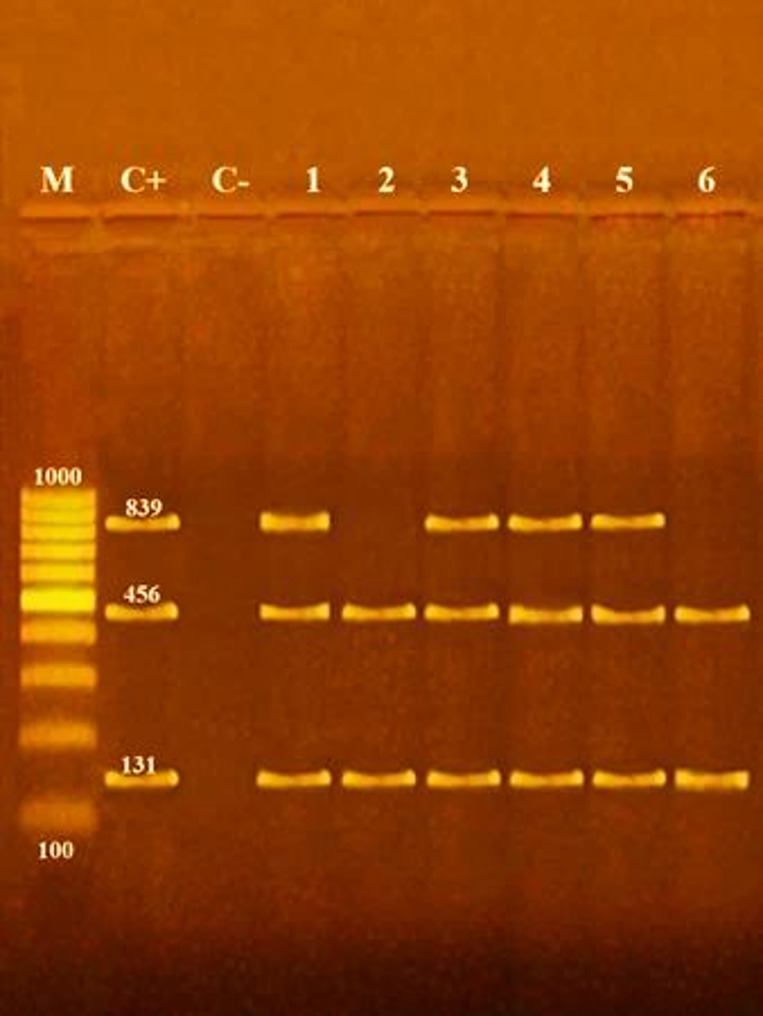
Agarose gel electrophoresis of multiplex PCR of *iap*(131 bp), *hylA* (456 bp) and *actA* (839 bp) genes for characterization of *L. monocytogenes* isolated from human. Lane M: 100 bp ladder as molecular size DNA marker. Lane C+: Control positive *L. monocytogenes* for *iap*,*hylA* and *actA* genes. Lane C-: Control negative. Lanes 1, 3, 4 & 5: Positive strains for *iap*,* hylA* and *actA *genes. Lanes 2 & 6: Positive *L. monocytogenes* strains for *iap *and*hylA* genes.

PCR amplification confirmed that all isolates carried the *iap* gene, while *hlyA* and *actA* were detected in 80% and 67% of isolates, respectively, Fig. (1 &2).

## Discussion

The present study investigated the prevalence, antimicrobial resistance patterns, serotype distribution, and virulence genes of *Listeria monocytogenes* isolated from food products, farm animals, and humans in Egypt. Our findings demonstrate a substantial public health risk posed by multidrug-resistant (MDR) *L. monocytogenes* strains circulating in the food chain and among occupationally exposed populations. The overall isolation rate of *Listeria* spp. (10.9%) in this study is comparable to findings from other Egyptian studies and international reports. Sotohy et al. (2024) reported prevalence rates between 8 and 12% in humans and ruminants from New Valley and Beheira Governorates [28], while Zakaria and Sabala (2024) found *L. monocytogenes* in 14.5% of poultry meat samples [29]. Similarly, Sołtysiuk et al. (2025) detected *Listeria* spp. in 9.7% of raw fish samples in Poland [30]. The surveillance of *L. monocytogenes* in Egyptian animals and humans is crucial for public health, as highlighted by a recent study [31].

The high prevalence observed in Kareish cheese (28.0%) and raw milk (22.0%) underscores the vulnerability of traditional dairy products to contamination. This is consistent with previous Egyptian studies, where artisanal cheese and raw milk were found to harbor *Listeria* at higher levels than pasteurized or industrially produced products [32]. The high prevalence in meat products is also of concern, as previously reported in Egyptian retail meat [33]. Internationally, soft cheeses have been linked to multiple listeriosis outbreaks in Europe and North America [5]. The relatively lower rate in yogurt (8.0%) may be attributed to the inhibitory effect of lactic acid and low pH, though the presence of *Listeria* in yogurt still reflects post-processing contamination risks, as the heat treatment during manufacturing should inactivate the pathogen. This highlights a critical public health failure in the handling process [34, 35]. Studies have also highlighted contamination along the milk-processing chain, emphasizing the need for robust decontamination practices, especially among small-scale retailers [36, 37]. The role of beneficial bacteria, such as cocci lactic acid bacteria in traditional Egyptian dairy products, is also being studied for their antimicrobial characteristics [38].

Animal fecal samples showed a relatively low prevalence (4.75%), similar to that reported by Matle et al. (2020) in Egyptian livestock [39]. This suggests that contamination at the farm level is lower than at post-harvest or processing stages, where cross-contamination is more likely. Nevertheless, the detection of *L. monocytogenes* in aborted fetuses (18.0%) highlights its role in reproductive failures, a finding consistent with Ahmed et al. (2023) in Upper Egypt [40]. The human carriage rate (17.0%) observed in this study is concerning, especially among farm workers (24.0%). Comparable occupational exposure was documented by Abou-Khadra and El-Azzouny (2024), who found similar prevalence rates among food sector workers in Egypt [41]. Such findings reinforce the notion that food handlers and agricultural workers may act as reservoirs and vectors for *Listeria* transmission into the food chain.

A major finding of this study is the high prevalence of antimicrobial resistance (AMR) among *L. monocytogenes* isolates. All isolates showed resistance to clindamycin and nalidixic acid, while high resistance rates to penicillin and ampicillin were also observed. Similar alarming resistance profiles were reported in Egyptian poultry and dairy isolates [42, 43]. Recent studies confirm this trend: Zakaria and Sabala (2024) found high MDR rates in poultry-associated *L. monocytogenes* [29], while Sotohy et al. (2024) documented comparable resistance patterns in ruminant and human isolates [28]. Globally, increasing resistance to β-lactam antibiotics such as penicillin and ampicillin is concerning, given that these drugs are considered first-line therapy for listeriosis [43]. The observation of similar resistance profiles between human and animal isolates suggests a high degree of horizontal gene transfer and a shared resistome. This points to a strong “One Health” link, where antibiotic resistance genes are readily exchanged between different environmental and host niches. The high resistance in human isolates, in particular, may be a direct consequence of antibiotic use in livestock, which then contaminates the food chain and subsequently human carriers. On the other hand, imipenem and amikacin remained effective in our study, consistent with international reports [2]. These results underscore the urgent need for prudent antibiotic use in livestock systems and for strengthening AMR surveillance in Egypt. Promisingly, alternative agents such as essential oils have shown potential to combat virulence and resistance in *L. monocytogenes*, offering a potential future direction for control [44].

Serotyping revealed that 4b was the dominant serotype across sources, a finding of major epidemiological significance. Serotype 4b is the most frequently implicated in severe outbreaks worldwide [45]. Its predominance in Egypt has also been reported by Badawy et al. (2022) in livestock-associated isolates [3]. The universal presence of the *iap* gene, combined with a high prevalence of *hlyA* and *actA*, demonstrates that the majority of isolates possessed key virulence determinants required for adhesion, invasion, and intracellular survival. Similar virulence gene distributions were reported in Egyptian isolates by Mahmoud et al. (2024) [46]. This is consistent with other local studies that have also characterized virulence genes in food and human isolates in Egypt [47]. This suggests that *L. monocytogenes* circulating in Egypt not only shows multidrug resistance but also retains strong pathogenic potential. The coexistence of multidrug resistance, virulence determinants, and the predominance of outbreak-associated serotypes (such as 4b) in food and human isolates represents a critical “One Health” concern. The high prevalence in dairy and environmental samples further emphasizes the role of the environment in the transmission cycle, highlighting the need for comprehensive surveillance beyond the food products themselves [48]. Contaminated dairy and meat products could serve as vehicles for transmission to humans, while occupationally exposed individuals could act as carriers contributing to cross-contamination.

## Conclusion

This study provides comprehensive insights into the epidemiology of *Listeria monocytogenes* in Egypt across food products, farm animals, and occupationally exposed humans. The detection of multidrug-resistant isolates, the predominance of outbreak-associated serotype 4b, and the presence of key virulence genes (iap, hlyA, actA) highlight the significant public health risks posed by *L. monocytogenes* in the Egyptian food chain. The high contamination rates observed in traditional dairy products such as Kareish cheese and raw milk emphasize the urgent need for improved hygienic practices during artisanal production and distribution. Furthermore, the widespread resistance to first-line antibiotics, particularly penicillin and ampicillin, underlines the necessity of prudent antibiotic use in veterinary and human medicine. A “One Health” approach integrating surveillance in humans, animals, and food products is essential for effective prevention and control strategies. Continuous monitoring, stricter food safety regulations, and antimicrobial stewardship programs should be prioritized to mitigate the risks associated with *L. monocytogenes* and safeguard public health in Egypt.

## Supplementary Information

Supplementary Material 1.

Supplementary Material 2.

## Data Availability

The datasets used and/or analyzed in the current study were not publicly published to preserve the privacy of the participants but are available upon reasonable request from the corresponding author.
