# Supplementary material for: Gene profiling and serotyping of multidrug-resistant Listeria monocytogenes isolated from humans, animals, and dairy products
Source: BMC Vet Res. 2025 Nov 22;21:702. doi: 10.1186/s12917-025-05138-4 (PMC12702151; doi:10.1186/s12917-025-05138-4)
Supplement: Supplementary file 1 — Supplementary Material 1. [file 12917_2025_5138_MOESM1_ESM.pdf]

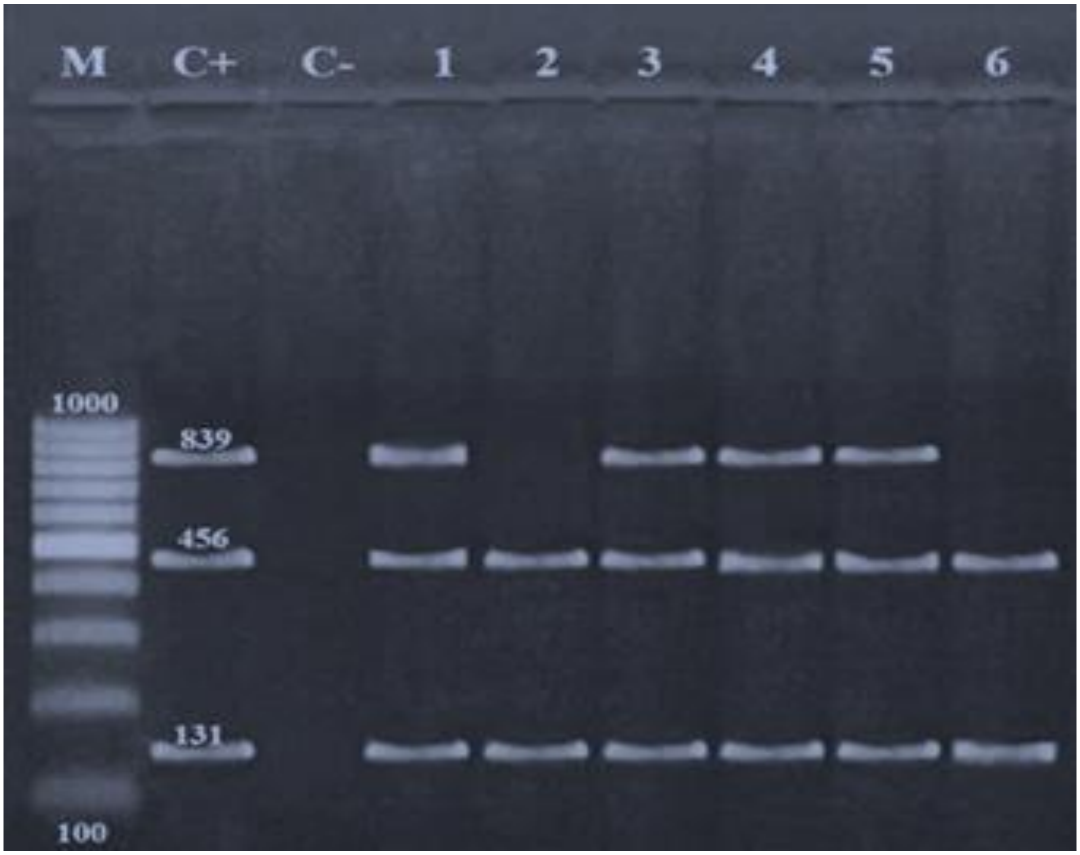


**Fig. (2).** Agarose gel electrophoresis of multiplex PCR of *iap* (131 bp), *hylA* (456 bp) and *actA* (839 bp) genes for characterization of *L. monocytogenes* isolated from human. **Lane M**: 100 bp ladder as molecular size DNA marker. **Lane C+:** Control positive *L. monocytogenes* for *iap*, *hylA* and *actA* genes. **Lane C-:** Control negative. **Lanes 1, 3, 4 & 5:** Positive strains for *iap, hylA* and *actA* genes. **Lanes 2 & 6:** Positive *L. monocytogenes* strains for *iap* and *hylA* genes.
