# Supplementary material for: Gene profiling and serotyping of multidrug-resistant Listeria monocytogenes isolated from humans, animals, and dairy products
Source: BMC Vet Res. 2025 Nov 22;21:702. doi: 10.1186/s12917-025-05138-4 (PMC12702151; doi:10.1186/s12917-025-05138-4)
Supplement: Supplementary file 2 — Supplementary Material 2. [file 12917_2025_5138_MOESM2_ESM.pdf]

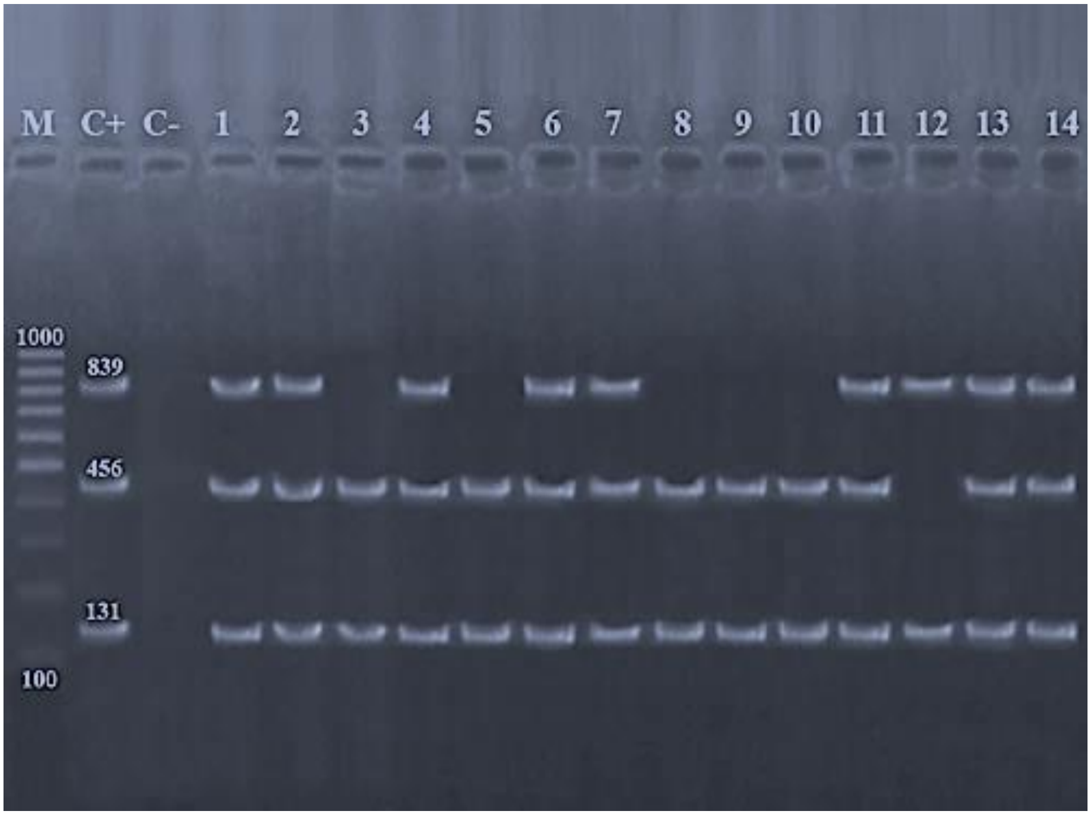

Fig. (1). Agarose gel electrophoresis of multiplex PCR of *iap* (131 bp), *hylA* (456 bp) and *actA* (839 bp) genes for characterization of *Listeria monocytogenes* isolated from animal sources. (Lane M: 100 bp ladder as molecular size DNA marker. **Lane C+:** Control positive *L.monocytogenes* for *iap*, *hylA* and *actA* genes. **Lane C-:** Control negative. **Lanes 1, 2, 4, 6, 7, 11, 13 & 14:** Positive strains for *iap*, *hylA* and *actA* genes. **Lanes 3, 5, 8, 9 & 10:** Positive *L. monocytogenes* strains for *iap* and *hylA* genes. **Lane 12:** Positive *L. monocytogenes* strain for *iap* and *actA* genes**).**
